# Gordon Holmes Syndrome Model Mice Exhibit Alterations in Microglia, Age, and Sex-Specific Disruptions in Cognitive and Proprioceptive Function

**DOI:** 10.1523/ENEURO.0074-23.2023

**Published:** 2024-01-19

**Authors:** Arlene J. George, Wei Wei, Dhanya N. Pyaram, Morgan Gomez, Nitheyaa Shree, Jayashree Kadirvelu, Hannah Lail, Desiree Wanders, Anne Z. Murphy, Angela M. Mabb

**Affiliations:** ^1^Neuroscience Institute, Georgia State University, Atlanta 30302, Georgia; ^2^Center for Behavioral Neuroscience, Georgia State University, Atlanta 30303, Georgia; ^3^Department of Nutrition, Georgia State University, Atlanta 30303, Georgia

**Keywords:** ataxia, cognitive, Gordon Holmes syndrome, microglia, RNF216, ubiquitin

## Abstract

Gordon Holmes syndrome (GHS) is a neurological disorder associated with neuroendocrine, cognitive, and motor impairments with corresponding neurodegeneration. Mutations in the E3 ubiquitin ligase *RNF216* are strongly linked to GHS. Previous studies show that deletion of *Rnf216* in mice led to sex-specific neuroendocrine dysfunction due to disruptions in the hypothalamic–pituitary–gonadal axis. To address RNF216 action in cognitive and motor functions, we tested *Rnf216* knock-out (KO) mice in a battery of motor and learning tasks for a duration of 1 year. Although male and female KO mice did not demonstrate prominent motor phenotypes, KO females displayed abnormal limb clasping. KO mice also showed age-dependent strategy and associative learning impairments with sex-dependent alterations of microglia in the hippocampus and cortex. Additionally, KO males but not females had more negative resting membrane potentials in the CA1 hippocampus without any changes in miniature excitatory postsynaptic current (mEPSC) frequencies or amplitudes. Our findings show that constitutive deletion of *Rnf216* alters microglia and neuronal excitability, which may provide insights into the etiology of sex-specific impairments in GHS.

## Significance Statement

Gordon Holmes syndrome (GHS) is a rare neurological disorder associated with neuroendocrine, cognitive, and motor impairments with corresponding neurodegeneration. Although mutations in *Rnf216* have been identified in male and female GHS individuals, it has yet to be tested if removal of *Rnf216* causes cognitive and motor dysfunction. This study characterizes motor and learning behaviors in male and female GHS model mice. We found that deletion of *Rnf216* does not disrupt motor function but does lead to sex-specific effects on proprioceptive and age-dependent changes in cognitive behaviors. Our study provides new insight into how disruptions in *Rnf216* contribute to the emergence of sex-specific phenotypes in individuals with GHS.

## Introduction

Neurological disorders (NDs) are a large group of nervous system-altering diseases that affect a large fraction of the population and encompass a wide variety of signs and symptoms. Gordon Holmes syndrome (GHS) is one example of a rare ND where individuals exhibit neuroendocrine abnormalities and motor dysfunction (Holmes, 1908, 1910; Neuhauser and Opitz, 1975; Seminara et al., 2002; Gonzalez-Latapi et al., 2021). GHS patients often have cognitive decline with signs of dementia as evaluated through cognitive diagnostic tests (Wechsler Adult Intelligence Scale-Revised China, Mini-Mental State Examination, and Montreal Cognitive Assessment; Margolin et al., 2013; Ganos et al., 2015; Alqwaifly and Bohlega, 2016; Mehmood et al., 2017; Calandra et al., 2019; Lieto et al., 2019; Chen et al., 2020). Disruptions in neuroendocrine, motor, and memory functions have been observed in GHS individuals, which are correlated with white and gray matter abnormalities in hippocampal, cortical, and cerebellar regions. GHS individuals also exhibit thinning of the corpus callosum and gliosis (Margolin et al., 2013; Ganos et al., 2015; Alqwaifly and Bohlega, 2016; Mehmood et al., 2017; Calandra et al., 2019; Lieto et al., 2019; Chen et al., 2020). Although GHS was documented in the early 1900s (Holmes, 1908), causative gene mutations were only recently discovered (Margolin et al., 2013; Shi et al., 2014; Synofzik et al., 2014). Recessive mutations in *CHIP/STUB1* and *RNF216* along with recessive digenic mutations in *RNF216* and *OTUD4* have been identified in individuals diagnosed with GHS (Margolin et al., 2013; Shi et al., 2014; Alqwaifly and Bohlega, 2016; Hayer et al., 2017; Calandra et al., 2019; Chen et al., 2022). *RNF216* mutations have also been reported in GHS-related syndromes that include Huntington-like disorder and 4H syndrome (Santens et al., 2015; Chen et al., 2020).

Functionally, *CHIP/STUB1*, *RNF216*, and *OTUD4* encode for classes of protein enzymes that are responsible for adding and removing an abundant post-translational tag called ubiquitin to other proteins. Ubiquitin is a 76 amino acid protein that is covalently attached to other proteins in the cell. This posttranslational modification regulates diverse biological processes that include signal transduction, protein degradation, cell cycle regulation, DNA repair, inflammation, and neural development (Hershko and Ciechanover, 1998; Mabb and Ehlers, 2010; Upadhyay et al., 2017; Mabb, 2021). This ATP-dependent reaction utilizes an enzymatic cascade consisting of a ubiquitin-activating enzyme (E1), a ubiquitin-conjugating enzyme (E2), and a ubiquitin ligase (E3). These three types of enzymes act in concert to attach diverse configurations of ubiquitin to substrate proteins (Komander and Rape, 2012; Zheng and Shabek, 2017). Protein ubiquitination can also be reversed by deubiquitinases (DUBs), which remove a variety of ubiquitin configurations from substrates (Komander et al., 2009). CHIP/STUB1 and RNF216 are both classified as E3 enzymes, whereas OTUD4 is characterized as a DUB. In GHS, missense mutations within the catalytic regions of *RNF216* and *CHIP/STUB1* decrease the ability of these E3s to ubiquitinate their targets (Shi et al., 2014; Husain et al., 2017). Cumulatively, these findings strongly imply that disruptions in the ubiquitination of substrates facilitated by RNF216 and CHIP/STUB1 most likely contribute to GHS pathogenesis.

In a previous study, constitutive *Rnf216* KO mice were found to have sex-specific alterations in reproductive phenotypes that were accompanied with altered microglia in the preoptic area of the hypothalamus (George et al., 2022). Given that GHS is a rare disorder with reported clinical differences in males and females, comorbidity of motor and learning impairments, and limited longitudinal tracking for pathogenesis, we monitored motor and cognitive functions in *Rnf216* male and female KO mice up to 1 year of age. Here, we found that KO females displayed abnormal limb clasping without any outstanding motor phenotypes in KO males and females. When evaluating cognitive function, *Rnf216* constitutive KO mice exhibited spatial and associative learning impairments that were age dependent. These alterations in learning and memory were associated with sex-specific changes in reactive microglia in the hippocampus and cortex. Although male and female KO mice had no changes in CA1 hippocampal miniature excitatory postsynaptic current (mEPSC) frequencies or amplitudes, KO males had a more negative resting membrane potential (RMP). Our findings show that deletion of *Rnf216* in mice leads to alterations in microglia and neuronal excitability, which may lead to impairments in select behavioral outputs.

## Materials and Methods

### Animals

Mice were kept in standard housing with littermates, provided with food and water *ad libitum*, and maintained on a 12 h light/dark cycle. All behavioral tests were conducted in accordance with the National Institutes of Health Guidelines for the Use of Animals. Mice were treated in accordance with the Animal Welfare and Ethics Committee (AWERB), and experiments were performed under the appropriated project licenses with local and national ethical approval. All experimental mice used in this study were housed separately by sex and in groups of 4–5 with balanced WT and KO genotypes. Sample sizes for behavior and immunohistochemistry experiments were calculated using variance from previous experiments to indicate power, in which statistical analysis for significance was set at 95%. All behavioral studies and isolation of body tissue for biochemical experiments were approved by the Animal Care and Use Committee.

### Generation of *Rnf216* knock-out mice

Embryonic stem (ES) cell clones were generated to target exons 4–5 of the *Rnf216* gene on mouse chromosome 5, which prevents the production of all isoforms (International Knockout Mouse Consortium). ES cell clones were injected into blastocytes and implanted into pseudopregnant mice. These mice were crossed to heterozygous mice for Flp recombinase to excise out the LacZ/neomycin cassette to obtain one *Rnf216* allele flanked by loxP (fl) sites to generate C57BL/6N-Rnf216<tm1c(EUCOMM)Wtsi>/Tcp (*Rnf216^wt/fl^*; Canadian Mouse Mutant Repository at the Hospital for Sick Children). Homozygous floxed conditional male mice (*Rnf216^fl/fl^*) were crossed with homozygous *CMV*-CRE*^+/+^* female mice (The Jackson Laboratory, JAX stock #006054) to allow the CRE to excise out exons 4 to 5, creating a dysfunctional gene. To breed out the CRE, we bred female offspring that were heterozygous for loxP and CRE (*Rnf216^wt/fl^::CMV-CRE^−/+^*) with WT males to generate heterozygous *Rnf216^−/+^* mice. Male *Rnf216^−/+^* mice were then bred with WT females to generate *Rnf216^−/+^* mice. *Rnf216^−/+^* male and female mice were then bred together to generate the experimental mice used for this study [*Rnf216^+/+^* (WT), *Rnf216^−/+^* (HET), and *Rnf216^−/−^* (KO)].

### Righting reflex

Mice were tested at ages postnatal day (P) 7 and P12 for developmental motor impairment. Briefly, mouse pups were separated from their mother and placed in a small paper box. One pup was removed from the box and placed in a supine position on a paper surface. Upon release of the mouse, the experimenter measured the time it took for the pup to return to a prone position (assessed by ensuring all four paws touched the ground surface). Pups were placed back in the box to rest for 1 min, and the procedure was repeated one additional time. Following the final time, the pup was immediately returned to its mother so as not to induce excessive stress.

### Grip strength

Mice were tested in grip strength for muscular strength at ages P60 and P365. Briefly, we measured the peak force applied by the forelimbs of mice using a grip strength meter (Ugo Basile). Mice were acclimated to the testing room 30 min prior to performing the test. To measure forelimb strength, we gently lowered the mouse onto the grid with its two forepaws. The investigator gently pulled back on the tail of the mouse, ensuring that the torso of the mouse remained horizontal and peak force was measured. The mouse was placed back in its home cage for at least 5 min to rest. The procedure was repeated two additional times, and the apparatus was cleaned with 70% ethanol between each mouse.

### Rotarod

Mice aged P65 and P370 were tested for balance, coordination, physical condition, and motor planning using the Rotarod apparatus (Ugo Basile). Tests were performed for 5 consecutive days, and mice were returned to standard housing daily after each day of testing. Briefly, mice were acclimated to the testing room for at least 30 min prior to performing the assay. Mice were placed horizontally on a rotating cylinder (rod) which was suspended 4–6 inches above the cage floor and spinning at 4 rpm. Thick bench paper was placed on the bottom of the rotarod to minimize stress if the mouse fell off. The rotarod speed was increased gradually (20 rpm/min for a maximum speed of 40 rpm), and the time (latency) it took for the mouse to fall off the apparatus was measured. Any mouse that fell off the apparatus was retrieved by the investigator and placed back in its home cage. For the first session (day 1), mice were given three trials with a 60 s rest in between each trial.

### Open field

Mice aged P62 and P367 were tested in the open field activity apparatus (Med Associates), which uses an array of infrared (IR) photo beams to measure locomotion and motor activity in a square arena. The apparatus consisted of three sets of matching IR photo beams that projected across the open field along three axes: *x*, *y*, and *z*. The software, Activity Monitor 7, detected when and where the photo beams were disrupted by the presence of the mouse. The software also breaks the field arena into zones, or areas of interest, depending on the experiment; data analysis displayed the measurements accordingly. Briefly, mice were acclimated to the testing room for at least 30 min prior to performing the assay. Mice were placed in the novel open field chamber for a total time of 15 min. After the assay, mice were returned to standard housing.

### Clasping

A separate cohort of mice was used to evaluate clasping to avoid any changes related to behavioral tasks. Mice from this cohort were tested for clasping at 3, 9, and 41 weeks of age. A portion of this cohort was used for brain harvesting and measurements of cerebellar weights. Mice were suspended in the air by their tail for two sessions, 30 s each. A score of 0 was defined as no clasping with both forelimbs and hindlimbs extended; a score of 1 was flexion of forelimbs without clasping of hind limbs; a score of 2 was assigned when forelimbs and hindlimbs were clasped.

### Barnes maze

Mice aged P70 and P375 were first habituated to the Barnes maze 2 d prior to testing. On the first day of habituation, each mouse was allowed to explore the maze for ∼30 s and then gently directed to a random hole (not used during testing). The mouse stayed in the hole for 1.5 min and then was returned to their home cage. The maze was sprayed with 70% ethanol between each mouse. One day prior to testing, the mice were given a chocolate-flavored pellet treat (Bio-Serv Supreme Mini-Treats) in their home cage. Each mouse was tested only once per day. Prior to testing sessions, mice were acclimated to the room 30 min. Before each session (including the testing session), the mice were held in an opaque cylinder in the middle of the maze before recording. Each session cutoff was set to 15 min. If the mouse did not find the exit hole during the 15 min, then the investigator guided the mouse into the hole. The learning task was a 10 day duration. On days 1–5 (training), the exit hole contained a chocolate treat to associate entry into the exit box as a positive and motivating experience. Once the session was completed, the mouse was held in a holding cage until all mice in the home cage were tested. On days 6–10 (learning), the chocolate-flavored treat was given in the home cage after each mouse in the cage was done testing. During the reversal phase on days 11–16 (reversal), the exit hole was rotated 180°, and the mice were still given a chocolate-flavored treat in the home cage after testing. Mice were excluded from the study if they did not enter the correct exit hole within the 15 min time limit by day 7. The scoring and criteria for search strategy was performed as previously described (Wall et al., 2018).

### Fear conditioning

Mice aged P90 and P395 were acclimated to the testing room for at least 30 min prior to performing the assay. On day 1 (conditioning), each mouse was placed in the testing chamber that contained a sound-attenuating box, and the mouse was allowed to explore for 2 min. Mice were then exposed to a 30 s tone (80 dB), followed by a 2 s scrambled foot shock (0.4 mA). Mice received two additional shock–tone pairings, 80 s between each pairing. Mice were then returned to a new, clean cage after testing. After all mice from one cage were tested, they were returned to the original home cage. On day 2 (context-dependent recall), mice were placed back in the original conditioning chamber for a test of contextual learning across a 5–6 min session. There was no shock or tone. On day 3 (cue-dependent recall), mice were placed back in the original conditioning chamber with new inserts attached to the walls of the chamber to provide a different environment. There was an auditory cue similar to day 1 without the shock in a 5–6 min session.

### Western blotting

Mouse tissue was thawed on ice and 300–500 µl of RIPA buffer was added. The tissue was then homogenized using sterile pestles. The samples were then centrifuged at 14,000 rpm for 15 min at 4°C, and the supernatant was collected for protein quantification. The protein concentration of the soluble fraction was measured using the Pierce 660 nM Protein Assay Kit (Thermo Fisher). The samples underwent SDS-polyacrylamide gel electrophoresis and were transferred on a nitrocellulose membrane (Bio-Rad) for 1 h at 70 mV. The blots were incubated overnight at 4°C with blocking buffer [Intercept (TBS) blocking buffer, Li-COR]. Membranes were then probed with the following primary antibodies prepared in a 1:1 ratio of TBST (1× TBS, 0.1% Tween 20) and blocking buffer solution containing a 1:1,000 dilution of 20% NaN_3_, rabbit polyclonal anti-RNF216 [LifeSpan Biosciences (amino acids 100–150), 1:1,000] and mouse anti-β-actin (GeneTex, 1:3,000), and then incubated overnight at 4°C. Membranes were washed three times in distilled water. The following secondary antibody dilutions were prepared in a 1:1 ratio of TBST and blocking buffer solution with 1:2,000 20% SDS, IRDye 680RD goat anti-mouse IgG (H + L; Li-COR, 1:20,000) and IRDye 800CW goat anti-rabbit IgG (H + L; Li-COR, 1:15,000), and were incubated for 1 h at room temperature. Membranes were washed two times in TBST and then two times with distilled water. Blots were imaged using the Odyssey CLx Imaging System (LI-COR) with a resolution of 169 µm, medium quality, and a 0 mm focus offset. Images were processed using the Gel Analysis tool in ImageJ using individual channels. Briefly, boxes were drawn around each band. Once the lanes were labeled and plotted, the area of the peaks was selected and measured. For each blot, proteins of interest were normalized to the Beta-ACTIN loading control.

### Microglia staining, imaging, and analysis

Male and female WT and KO mouse brains were sectioned at 40 µm. Sections for each mouse were selected based on stereological matching using the mouse brain stereotaxic coordinate reference atlas. Samples were then blinded and processed as follows: Free-floating sections were rinsed with 3% hydrogen peroxide two times for 7 min each to remove any endogenous peroxidases and then washed six times in 1× KPBS (in mM, 1.6 NaCl, 0.4 K_2_HPO_4_, and 0.09 KH_2_PO_4_ dissolved in ddH_2_O to make a 10× solution and diluted to 1×) at room temperature. Sections were then incubated overnight at room temperature in 1× KPBS containing 1.0% Triton and 1:50 K dilution of goat anti-Iba1 (Novus Biologicals). Sections were then washed 10 times with 1× KPBS before incubation in 1× KPBS containing 0.4% Triton and 1:600 dilution of donkey anti-goat biotin-SP (Jackson ImmunoResearch) for 1 h at room temperature. Sections were washed five times in 1× KPBS before being incubated in avidin–biotin–peroxidase complex (1:10, ABC Elite Kit, Vector Laboratories) for 1 h at room temperature. After rinsing sections three times in 1× KPBS and three times in 0.175 M sodium acetate buffer, Iba1 immunoreactivity was visualized with nickel sulfate enhanced 3,3′-diaminobenzidine (DAB) solution (0.2 mg/ml) containing 0.08% hydrogen peroxide in 0.175 M sodium acetate buffer. Sections were incubated in the DAB solution for 15 min before rinsing three times with 0.175 M sodium acetate buffer followed by three times in 1× KPBS. Sections were then mounted onto gelatin-subbed slides, air-dried, then dehydrated in a graded series of alcohols, and cleared with xylenes. Slides were then coverslipped with Permount mounting media. Bright-field images of the dorsal hippocampus and S1 cortex were acquired with the researcher blinded to condition on a Keyence BZ-X700 (Keyence) microscope using a 20× Plan Apochromat 0.75 N.A. objective with a *z*-step size of 1 µm. For densitometry, background subtraction and thresholding were first performed on the images using FIJI/ImageJ (National Institutes of Health). Cell count analysis was performed using CellProfiler 4.2.1 (Broad Institute; Carpenter et al., 2006). As the morphology of multiple microglia were analyzed from one mouse, the hierarchical bootstrapping and permutation tests were employed as they do not require the assumption of sample independence (Saravanan et al., 2020). The area of microglia soma in the S1 cortex and dorsal hippocampus was measured on maximum intensity projection images by using the freehand selection tool in FIJI ImageJ2 version 2.9.0/1.53t. Normal distribution scores for the mean were then analyzed by hierarchical bootstrapping using the ClusterBootstrap package program (Deen and de Rooij, 2020) in R Software version 4.2.3 (Free Software Foundation). For quantification of microglia branching, microglia were traced using the Simple Neurite Tracer (SNT) plugin for FIJI ImageJ2. The intersections of skeletonized microglia were then measured using the Sholl analysis plugin in FIJI ImageJ2 with the concentric circle set to 1 µm increments. The area under the curve (AUC) for each microglia was then assessed using the ClusterBootstrap package program in R. The permutation testing was set at 1,000 random shufflings, which allowed us to determine the robustness of the estimated means. Data were visualized as a probability density function implemented in MS Excel 2023 version 16.75.2 (Microsoft). Representative microglia reconstructions were created from maximum intensity projection images using the SNT plugin tool fill option. Constructed images were exported and cropped using identical scales.

### Electrophysiology

Male and female WT and *Rnf216* KO (P28) mice were sacrificed, and hippocampal slices were prepared for electrophysiological recordings. After decapitation, the brains were dissected out quickly and immediately immersed in oxygenated (95% O_2_ and 5% CO_2_) ice-cold artificial cerebrospinal fluid (aCSF) containing the following (in mM): 126 NaCl, 2.5 KCl, 2.4 CaCl_2_, 1.2 NaH_2_PO_4_, 1.2 MgCl_2_, 11.1 glucose, 21.4 NaHCO_3_. Hippocampal transverse slices (300 µm thick) were cut by using a Leica VT1000S Vibratome (Leica Microsystems). The brain slices were transferred to a holding chamber filled with oxygenated aCSF and kept at room temperature (25°C) for 1 h before recording. For recording, a single brain slice was transferred to the recording chamber and continuously perfused with oxygenated aCSF maintained at 30°C.

All recordings were performed using an Axopatch 700B amplifier and a Digidata 1440A data acquisition system (Molecular Devices). Whole-cell voltage-clamp recordings of mEPSCs were performed on an Olympus upright microscope (BX51WIF) with a 40× water immersion objective. The holding potential of all neurons was −70 mV. Recordings were filtered at 10 kHz and digitized at 10 kHz. The electrode internal solution contained the following (in mM): 120 K-gluconate, 10 KCl, 2 MgCl_2_, 10 HEPES, 0.1 CaCl_2_, 1 EGTA, 3 MgATP, and 0.5 NaGTP, pH 7.2 (with KOH). Only recording pipettes with a resistance of 2–3 MΩ after being filled with internal solution were used. Synaptic blockers APV (25 µM), picrotoxin (100 µM), and action potential blocker tetrodotoxin (1 µM TTX) were added in aCSF during the recordings. Series resistance was monitored, and the cells were discarded when the series resistance increased >15%. The liquid junction was −8 mV detected from the intracellular solution and subtracted from the membrane potential values in the final result. The mEPSCs were analyzed using MiniAnalysis with threshold events set at 5 pA. Electrophysiology recordings and data analysis were conducted with the experimenter blinded to genotype.

### Statistical analysis

Statistical analyses applied were the post hoc Student’s *t* test, one-way ANOVA, chi-square test (for categorical limb clasping data), Pearson’s correlation, and two-way and three-way ANOVA with multiple comparisons. For *k* = 2, data were analyzed using Student’s *t* test. When *k* > 2, data were analyzed for significant main effects of genotype, age, and sex using ANOVA. When time (trials) was a factor or there were missing values, data were analyzed using a mixed model ANOVA. If no significant main effect of sex was observed, then data were collapsed to increase power. Sidak’s or Tukey’s tests were used for comparing group means only when a significant *F* value was determined. For all comparisons, significance was set at *p* < 0.05. Data presented in figures and tables are means (± SEM). See Extended Data Table 1 for statistical reporting table.

10.1523/ENEURO.0074-23.2023.t1-1 Extended Data 1 R program file code used for analysis of microglia. Download Data 1, ZIP file.

### Code accessibility

The code for microglia normal distribution scores by hierarchical bootstrapping is provided as Extended Data and is freely available online at https://github.com/mabblab/Microglia-analysis. Code was executed on a MacBook Air running the macOS Ventura 13.4.1 with hardware specifications of 8 GB of RAM and a 1.6 GHz Dual-Core Intel Core i5 Processor.

## Results

### No gross motor impairments in *Rnf216* KO mice

**Figure 1.**
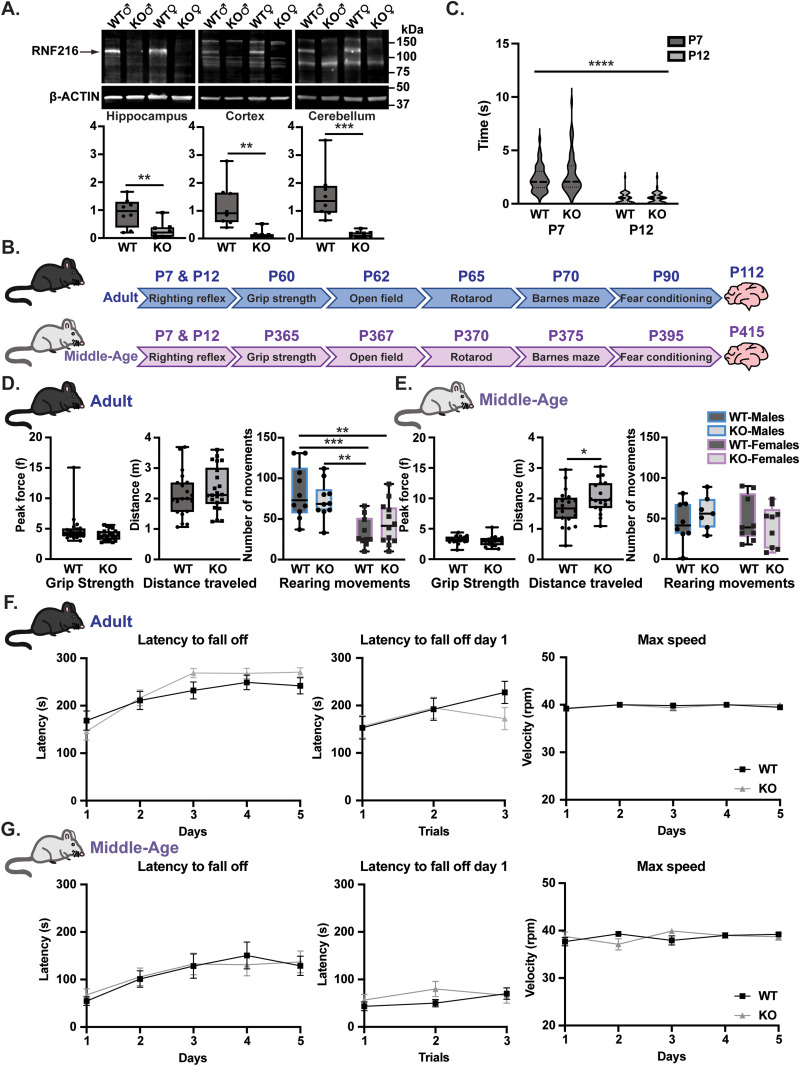
Neuromuscular and motor functions are unaffected in adult and middle-aged *Rnf216* KO mice. ***A***, Top, Representative Western blots for RNF216 in male and female *Rnf216^+/+^* (WT) and *Rnf216^−/−^* (KO) mice. Bottom, RNF216 protein levels in WT and KO mice. RNF216 values were normalized to ACTIN. For the hippocampus, *t*_(14)_ = 3.583, **p = 0.0030; cortex, *t*_(14)_ = 3.676, **p = 0.0025; and cerebellum, *t*_(14)_ = 4.337, ***p = 0.0007. Unpaired *t* test. *N *= 4 per genotype/sex for each brain region. Data are represented as box and whisker plots. ***B***, Behavior battery timeline for adults (P60; top) and middle-aged (P365) mice (bottom). ***C***, No differences in righting reflex in KO mice. *F*_Time(1,96)_ = 210.8, ****p < 0.0001; *F*_Genotype(1,96)_ = 1.943, *p* = 0.1666; *F*_Time*Genotype(1,96)_ = 2.261, *p* = 0.1359. Two-way ANOVA. *N *= 49 mice per genotype. Data are represented as a violin plot. ***D***, Left, Average peak force of grip strength for adult mice. *t*_(40)_ = 1.684, *p* = 0.0999. Middle, Total distance traveled in adult mice in an open field. *t*_(40)_ = 0.7746, *p* = 0.4431. Unpaired *t* test. *N *= 20 for WT and *N *= 23 for KO. Right, Total rearing movements in adult in an open field. *F*_Sex(1,38)_ = 23.80, ****p < 0.0001; *F*_Genotype(1,38)_ = 0.004722, *p* = 0.9456; *F*_Sex*Genotype(1,38)_ = 1.721, *p* = 0.1974. Post hoc: WT-Males vs WT-Females, ***p = 0.0007; WT-Males vs KO-Females, **p = 0.0067; WT-Females vs KO-Males, **p = 0.0078. Two-way ANOVA with Tukey’s multiple comparisons. *N *= 10–12 mice per sex/genotype. Data are represented as box and whisker plots. ***E***, Left, Average peak force of grip strength in middle-aged mice *t*_(31)_ = 0.7981, *p* = 0.4309. Middle, Total distance traveled in middle-aged mice in an open field. *t*_(31)_ = 2.081, *p = 0.0457. Unpaired *t* test. *N *= 17 for WT and *N *= 16 for KO. Right, Total rearing movements in adult in an open field. *F*_Sex(1,29)_ = 0.5386, *p* = 0.4689; *F*_Genotype(1,29)_ = 0.05047, *p = 0.8238; *F*_Sex*Genotype(1,29)_ = 1.199, *p* = 0.2826. Two-way ANOVA. *N *= 7–9 mice per genotype/sex. Data are represented as box and whisker plots. ***F***, No genotypic differences in adult male and female KO mice in latency to fall off the rotating rod (left), latency to fall off the rotating rod for each trial on day 1 (middle), or maximum velocity (right). *N *= 20–22 per genotype. ***G***, No significant differences in middle-aged male and female KO mice. *N *= 16–17 per genotype. Error bars measured as ±SEM in ***F*** and ***G***.

*Rnf216* knock-out (KO) mice have reproductive impairments with KO males having reduced testicular weights and females displaying alterations in estrus cycling (George et al., 2022). In addition to these neuroendocrine abnormalities, GHS individuals with mutations predicted to be deleterious for *RNF216* also exhibit ataxia and dementia with corresponding cortical and cerebellar atrophy (Margolin et al., 2013; Alqwaifly and Bohlega, 2016; Calandra et al., 2019). However, there are no studies that have established a role for *Rnf216* in motor function and learning, which are core phenotypes that are disrupted in individuals with GHS (Holmes, 1908; Margolin et al., 2013). As expected, we found a significant decrease in RNF216 in the hippocampus, cortex, and cerebellum in male and female *Rnf216* KO mice (Fig. 1*A*).

To establish a role for *Rnf216* deletion on GHS-related phenotypes, we devised a battery of behavior tests to evaluate motor and memory function in adult and middle-aged KO mice (Fig. 1*B*). Righting reflexes were found to be normal in P7 and P12 KO males and females suggesting that motor development was not grossly impaired in developing mice (Fig. 1*C*). We next measured peak force using the grip strength assay but found no significant differences (Fig. 1*D*,*E*, left), indicating that KO mice do not have deficiencies in neuromuscular strength. Based on these data, we sought to determine if KO mice had alterations in motor learning and coordination. We measured the total distance traveled and rearing movements in an open field test in adult and middle-aged mice (Fig. 1*D*,*E*, middle and right). Although there was no significant difference in the distance traveled in adult mice (Fig. 1*D*, middle), there was a sex-dependent reduction in the number of rearing movements in adult female mice (Fig. 1*D*, right). Middle-aged KO mice had an increase in the distance traveled in the center of the open field (Fig. 1*E*, middle), but there were no significant differences in the number of rearing movements between sex or genotype (Fig. 1*E*, right). Next, we measured another metric of motor function using the accelerating rotarod but found no differences in the latency to fall or maximum speed in adult and middle-aged KO mice (Fig. 1*F*,*G*).

**Figure 2.**
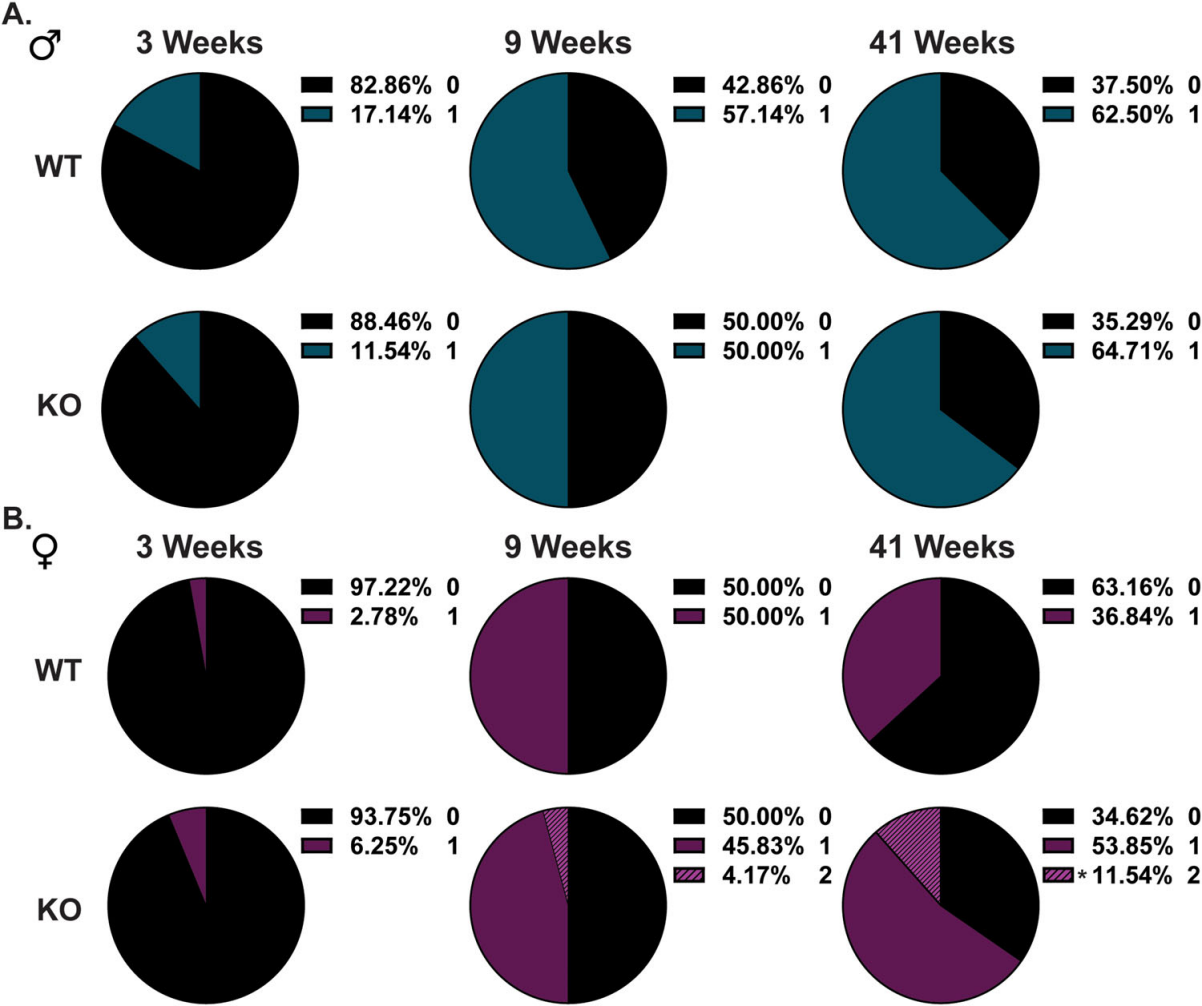
Emergence of abnormal limb-clasping in *Rnf216* female KO mice. ***A***, Pie charts of limb-clasping in WT- and KO-Males. Limb-clasping was scored based on the following parameters: 0, no clasping (black); 1, forelimb clasping only (teal); 2, forelimb and hindlimb clasping. ***B***, Pie charts of limb-clasping in WT- and KO**-**Females. Limb-clasping was scored based on the following parameters: 0, no clasping (black); 1, forelimb clasping only (purple); 2, forelimb and hindlimb clasping (striped purple). KO-Females began to show hindlimb clasping at 9 and 41 weeks. At 41 weeks for females, [*χ*^2^ (1, *N *= 45) = 4.724; *p *= *0.0297; chi-square for trend]. For 3 weeks, *N *= 35–36 for WT and 26–32 for KO; for 9 weeks, *N *= 22–31 for WT and 18–24 for KO; and for 41 weeks, *N *= 19–24 for WT and 17–26 for KO mice per sex/genotype.

Because cerebellar ataxia mouse models are known to display abnormal limb-clasping reflexes (Lalonde and Strazielle, 2011; Kojic et al., 2018), we measured the limb-clasping reflex in adult and middle-aged mice. Under normal conditions, adult mice splay out their forelimbs and hindlimbs away from their abdomen when held upside-down by their tail. However, limb reflex abnormalities result in the clasping of the forelimbs and hindlimbs when held upside-down. Limb-clasping was scored in WT and KO mice at different developmental ages that included weaning (3 weeks), young adult (9 weeks), and middle-aged (41 weeks). Although there was no significant difference in KO males (Fig. 2*A*), a small percentage (4.17%) of KO females had abnormal forelimb and hindlimb clasping that appeared to emerge as early as 9 weeks and increased at 41 weeks of age (11.54%; Fig. 2*B*). The onset of abnormal clasping in females could be related to reductions in cerebellar volume. However, we did not find any genotypic differences in cerebellar brain weights in 16- and 52-week-old male and female KO mice (Table 1). Taken together, these findings indicate that motor learning and coordination are intact in male and female *Rnf216* KO mice up to 1 year of age. However, given the limb-clasping phenotype, a subset of female *Rnf216* KO mice may have compromised proprioception.

**Table 1.** Cerebellar weights in 16 and >52 week mice

Cerebellar weights	16 weeks	*n*	*p* value	>52 weeks	*n*	*p* value
*Rnf216* global knock-out	WT = 20.0 ± 0.48 KO = 21.2 ± 0.71	WT = 18 KO = 11	0.16	WT = 21.0 ± 0.82 KO = 20.5 ± 0.82	WT = 18 KO = 25	0.70

There were no significant differences. *N *= 11–25 mice per genotype. Cerebellar weights were normalized to brain weights. Error bars are ± SEM.

### Middle-aged *Rnf216* KO mice reveal nuances in hippocampal-dependent learning and memory

**Figure 3.**
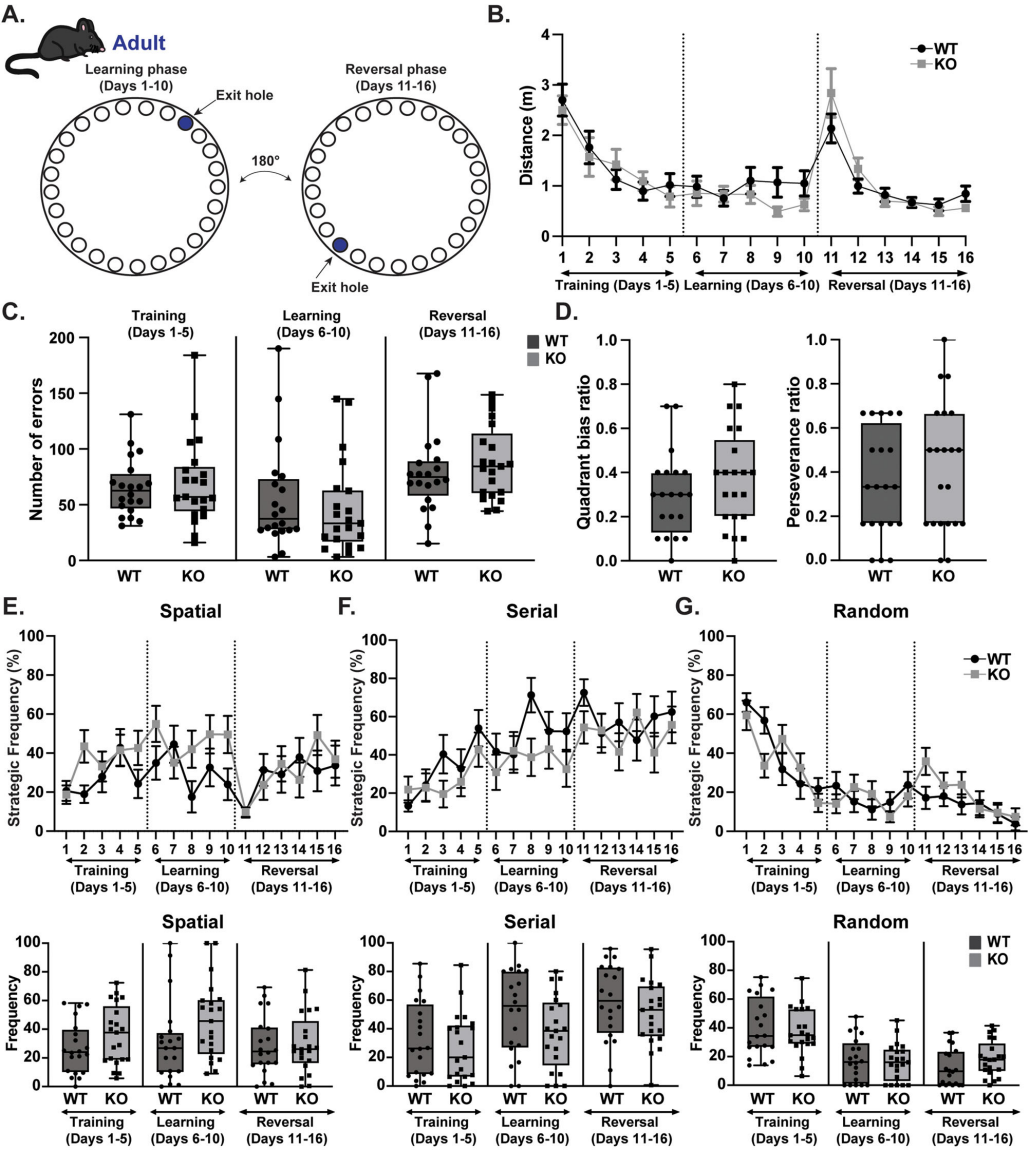
Lack of spatial learning deficits in adult *Rnf216* KO mice. ***A***, Schematic of Barnes maze task to evaluate spatial and reversal learning. Learning phase consisted of training (days 1–5) followed by assessments of learning (days 6–10). During the reversal phase (days 11–16), the exit hole was rotated 180°. ***B***, Adult (∼P70) WT and KO mice show no difference in total distance traveled on the maze before finding the exit hole. ***C***, No difference in the number of errors during the training, learning, or reversal phase. ***D***, Left, No differences in quadrant bias ratio. Right, no difference in perseverance ratio in KO mice. ***E***, Top, No differences in spatial strategy during each day across phases. Bottom, No differences in spatial strategy consolidated for each phase. ***F***, Top, No differences in serial strategy during each day across phases. Bottom, No differences in serial strategy consolidated for each phase. ***G***, Top, No differences in random strategy during each day across phases. Bottom, No differences in random strategy consolidated for each phase. *N *= 20 for WT and *N* = 21 for KO. Error bars are represented as ±SEM on top graphs in ***E***–***G*** and as box and whisker plots on bottom graphs.

Because GHS individuals with *RNF216* mutations exhibit dementia, we reasoned that *Rnf216* KO mice would develop impairments in spatial learning and memory. To explore the role of RNF216 in hippocampal-dependent spatial learning, we used the Barnes maze task (Eales et al., 2014). Mice were tested for 16 consecutive days to evaluate training (days 1–5), learning (days 6–10), and reversal learning (days 11–16; Fig. 3*A*). In both WT and KO mice, we found that the distance traveled significantly decreased during the training and learning phases (days 1–10) and during the reversal phase (days 11–16), indicating that all mice were able to learn the location of the exit hole. Evaluation of distance traveled in each phase did not reveal significant differences in sex or genotype (Fig. 3*B*). Next, we measured the number of errors performed by each mouse during training, learning, and reversal but found no sex- or genotype-specific differences (Fig. 3*C*). We also calculated the quadrant bias ratio as a measure of the frequency of visitation to the quadrant containing the exit hole during the training/learning phases (days 1–10) but also found no significant differences (Fig. 3*D*, left). Finally, we calculated the perseverance ratio as a reflection of the frequency of visitation to the previous location of the exit hole during the reversal phase but again found no significant differences (Fig. 3*D*, right). The employment of specific strategies to evaluate efficiency can also be utilized in the Barnes maze task. As described previously, we divided navigation strategies into three different categories: spatial, serial, and random (Wall et al., 2018). When separated into these different phases, we found no sex- or genotype-specific differences in strategy selection across phases (Fig. 3*E–G*).

**Figure 4.**
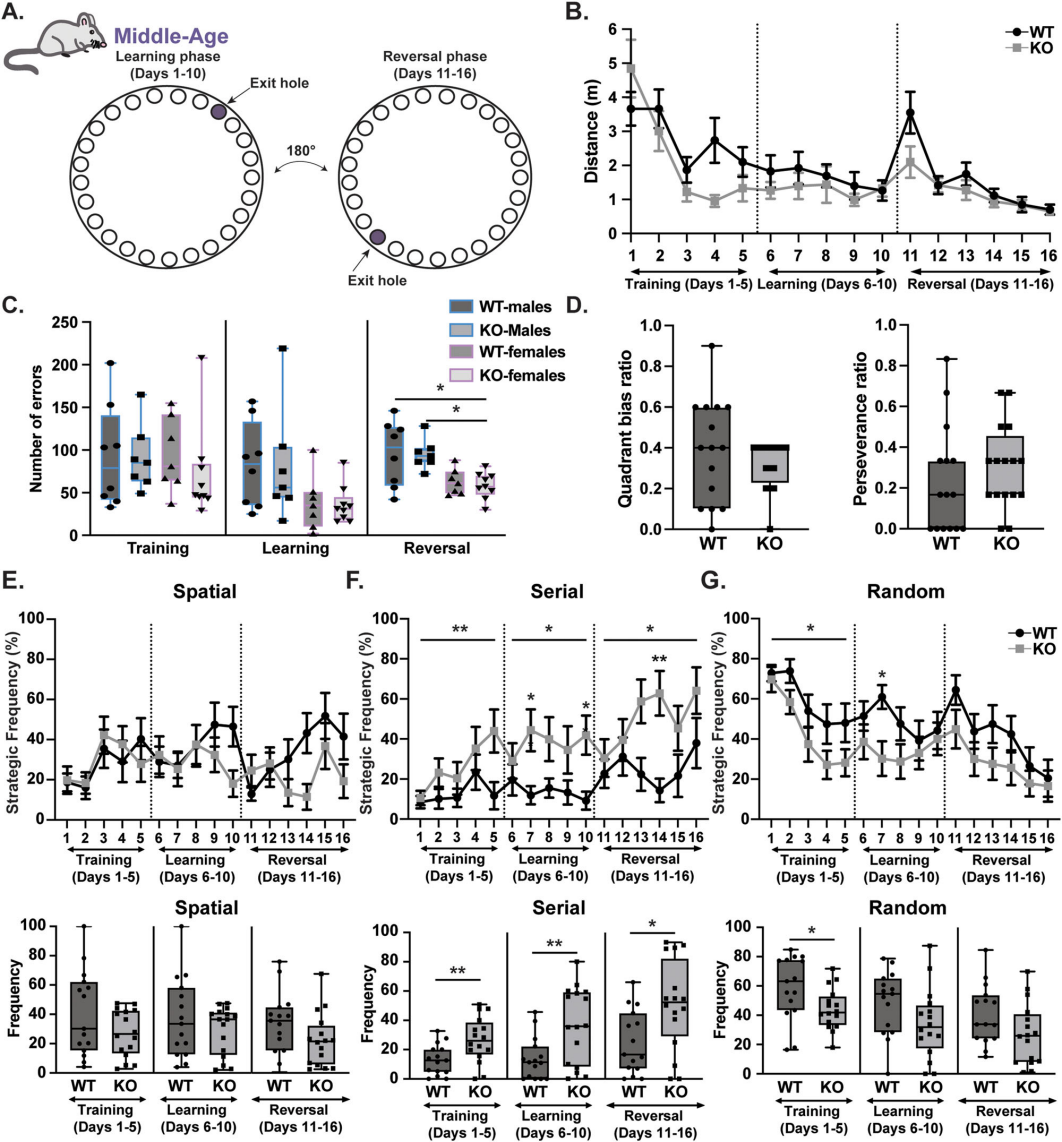
Altered search strategies in middle-aged *Rnf216* KO mice. ***A***, Schematic of Barnes maze task to evaluate spatial and reversal learning. Learning phase consisted of training (days 1–5) followed by assessments of learning (days 6–10). During the reversal phase (days 11–16), the exit hole was rotated 180°. ***B***, Middle-aged (∼P375) WT and KO mice show no difference in total distance traveled on the maze before finding the exit hole. ***C***, No genotypic differences in the number of errors in both males and females during training, learning, and reversal phase. There were sex differences during the reversal phase. KO-Males have a higher number of errors than KO-Females. *F*_Genotype(1,27)_ = 0.09622, *p* = 0.7588; *F*_Sex(1,27)_ = 16.42, ***p = 0.0004; *F*_Sex*Genotype(1,27)_ = 0.04985, *p* = 0.8250. Post hoc: WT-Males vs KO-Females, *p = 0.0156; KO-Males vs KO-Females, *p = 0.0239. Two-way ANOVA with Tukey’s multiple comparisons. ***D***, Left, No differences in quadrant bias ratio. Right, No difference in perseverance ratio. ***E***, Top, No differences in spatial strategy during each day across phases. Bottom, No differences in spatial strategy consolidated for each phase. ***F***, Top, Differences in serial strategy during each phase. Training: *F*_Time(2.770,79.62)_ = 2.583, *p* = 0.0637; *F*_Genotype(1,29)_ = 8.306, **p = 0.0074; *F*_Time*Genotype(4,115)_ = 1.121, *p* = 0.3502; learning: *F*_Time(3.436,99.63)_ = 0.1508, *p* = 0.9463; *F*_Genotype(1,29)_ = 8.537, **p = 0.0067; *F*_Time*Genotype(4,116)_ = 0.9341, *p* = 0.4468. Post hoc: day 7, *p = 0.0482; day 10, *p = 0.0309; reversal: *F*_Time(4.011,116.3)_ = 1.960, *p* = 0.1050; *F*_Genotype(1,29)_ = 6.954, *p = 0.0133; *F*_Time*Genotype(5,145)_ = 1.849, *p* = 0.1070. Post hoc: day 14, **p = 0.0050. Two-way ANOVA with Sidak’s multiple comparisons. Bottom, differences in serial strategy consolidated for each phase. Training: *t*_(29)_ = 2.838, **p = 0.0082; learning: *t*_(29)_ = 2.984, **p = 0.0057; reversal: *t*_(29)_ = 2.648, *p = 0.0130. Unpaired *t* test. ***G***, Top, Differences in random strategy during the training phase. *F*_Time(3.475,99.90)_ = 11.99, ****p < 0.0001; *F*_Genotype(1,29)_ = 5.205, *p = 0.0300; *F*_Time*Genotype(4,115)_ = 0.5664, *p* = 0.6875. Mixed-effects model. Bottom, Differences in random strategy during the consolidated training phase. *t*_(29)_ = 2.252, *p = 0.0320. Unpaired *t* test. *N* = 15 for WT and *N* = 16 for KO.

Given the lack of phenotype in adult KO mice, we next measured learning phenotypes in middle-aged mice (>1 year). Although there were no genotypic differences in KO males or females in total distance traveled, quadrant bias ratio, or perseverance ratio (Fig. 4*A–D*), there were sex-specific differences in the number of errors selectively during reversal with a significant effect found between male and female KO mice (Fig. 4*C*). Upon separating the assay into time blocks of phases, sex differences were not observed in strategy usage between groups (Fig. 4*E–G*, Extended Data Table 1). However, there was a main effect of genotype on serial and random strategy usage (Fig. 4*F*,*G*). *Rnf216* KO mice had a significant increase in using the serial strategy across training, learning, and reversal phases with a reduction in the random search strategy during the training phase. These findings show that upon aging, *Rnf216* KO mice selectively adopt alternative search strategies in a hippocampal-dependent spatial task.

**Figure 5.**
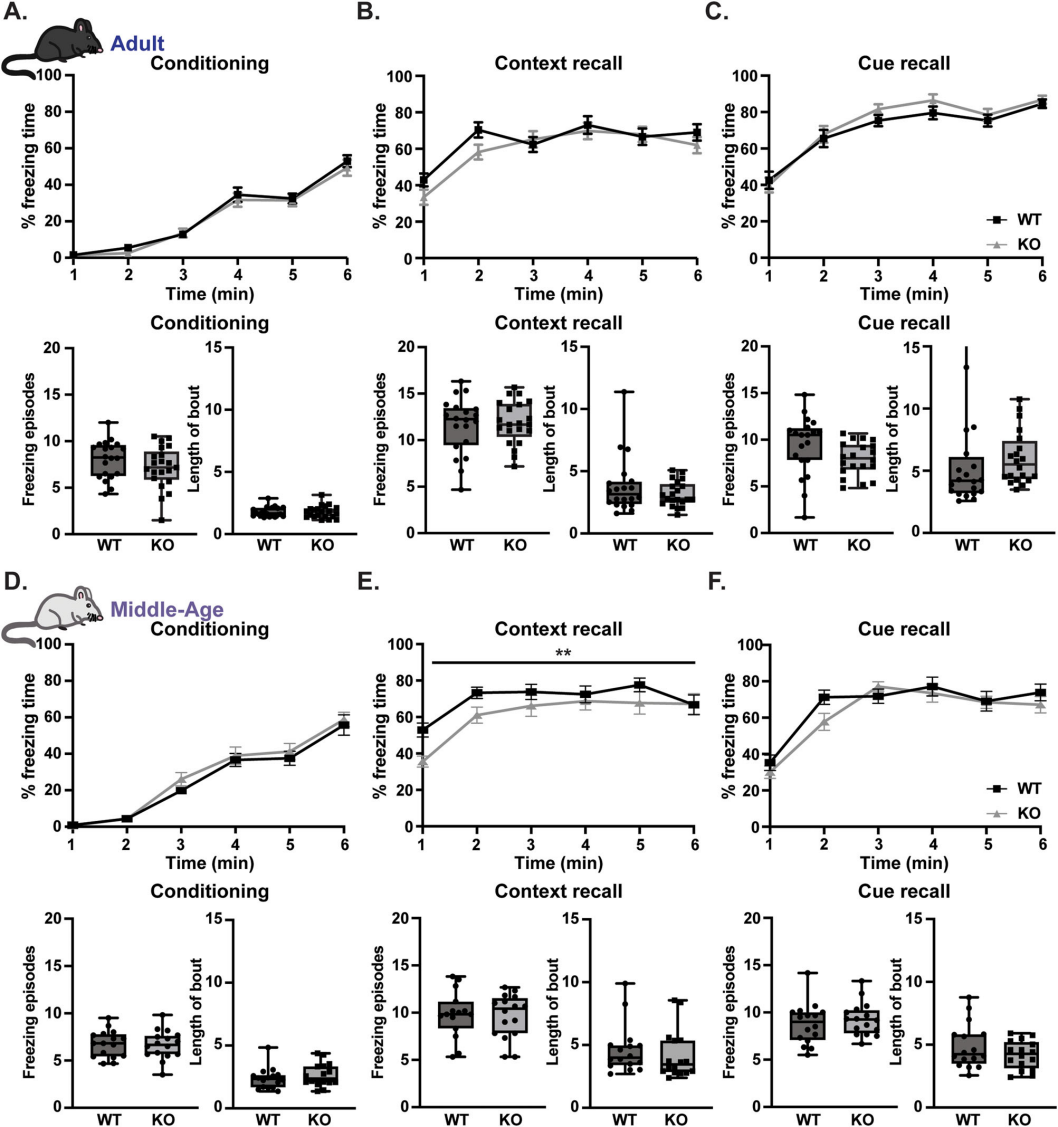
Decreased context recall in middle-aged *Rnf216* KO mice. ***A***, No differences in time spent freezing during conditioning on day 1 in adult WT and KO mice or average number of freezing episodes (bottom left) or length of bout (bottom right). ***B***, No differences in time spent freezing during context recall on day 2 in adult WT and KO mice or average number of freezing episodes (bottom left) or length of bout (bottom right). ***C***, No differences in time spent freezing during cue recall on day 3 in adult WT and KO mice or average number of freezing episodes (bottom left) or length of bout (bottom right). *N* = 10–11 mice per sex/genotype. ***D***, No differences in time spent freezing during conditioning on day 1 in aged WT and KO mice or average number of freezing episodes (bottom left) or length of bout (bottom right). ***E***, Middle-aged KO mice spent less time freezing during context recall on day 2. *F*_Time(4.038,121.1)_ = 12.15, ****p < 0.0001; *F*_Genotype(1,30)_ = 5.380, *p = 0.0274; *F*_Time*Genotype(5,150)_ = 1.089, *p* = 0.3690. Two-way ANOVA with Sidak’s multiple comparisons. There were no differences on average number of freezing episodes (bottom left) or length of bout (bottom right). ***F***, No differences in time spent freezing during cue recall on day 3 in middle-aged WT and KO mice or average number of freezing episodes (bottom left) or length of bout (bottom right). *N *= 7–9 mice per genotype/sex. Top, Error bars are represented as ±SEM. Bottom, Data are represented as box and whisker plots.

In addition to testing hippocampal-dependent spatial memory, we evaluated another type of learning that included associative learning through fear conditioning. In our fear conditioning task, mice were given three pairs of a tone associated with a shock (day 1). On day 2, mice were tested for context recall (same environment), and on day 3, mice were tested for cue recall (different environment + tone). Although there were no differences in time spent freezing, number of freezing episodes, or length of bout during conditioning, context recall, or cue recall in adult male and female KO mice (Fig. 5*A–C*), middle-aged KO mice had an overall decrease in freezing time during context recall (Fig. 5*E*).

**Figure 6.**
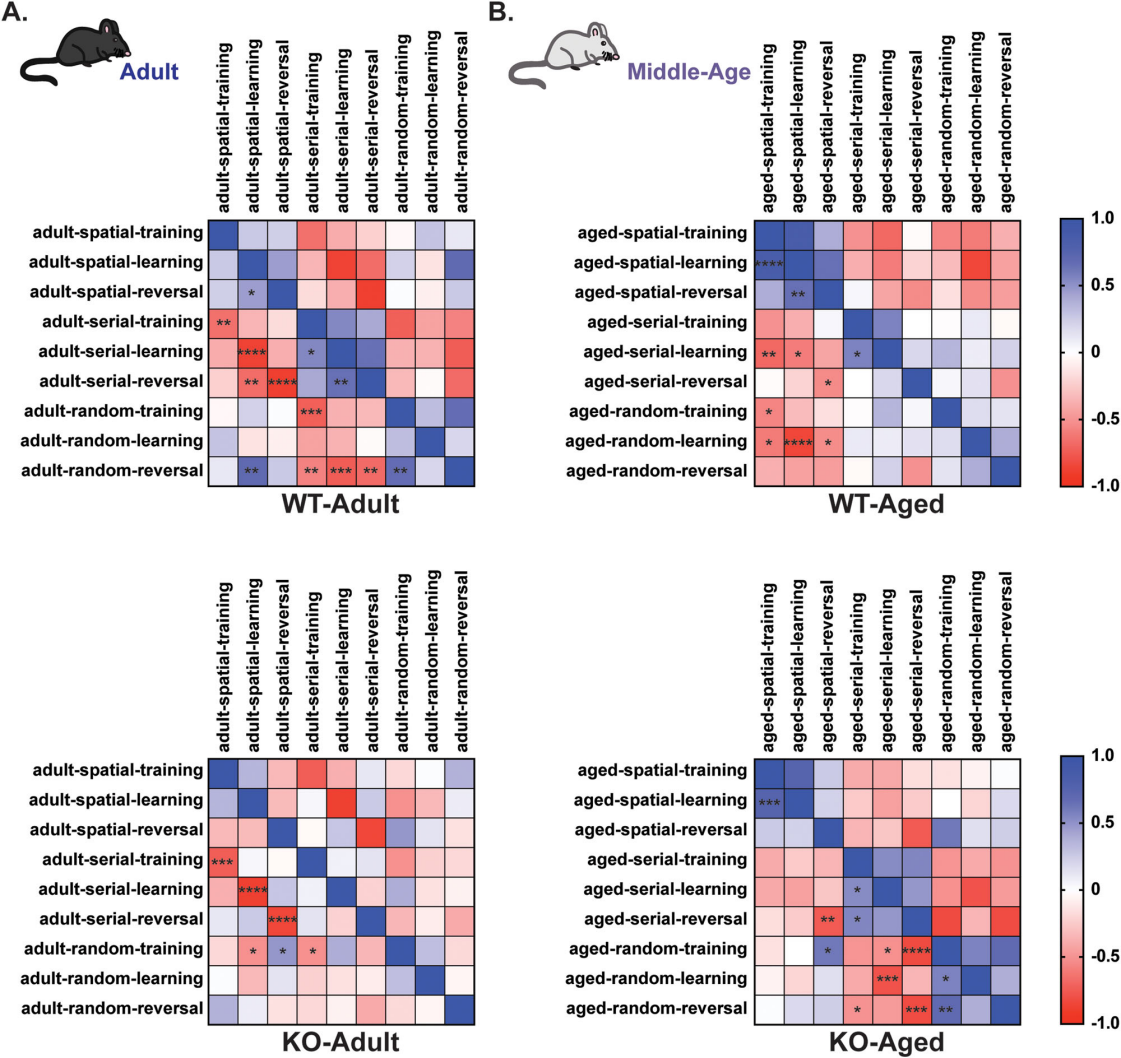
Middle-aged *Rnf216* KO mice exhibit search strategies that are more tuned to adult WT mice. ***A***, Pearson’s correlation in adult WT (top) and KO (bottom) mice using behavioral output parameter-related strategy search in the Barnes maze. ***B***, Pearson’s correlation in middle-aged WT (top) and KO (bottom) mice using behavioral output parameters related to open field, strategy search in the Barnes maze. *p < 0.05, **p < 0.005, ***p < 0.0005, ****p < 0.00005.

We next compared Barnes maze strategy behavioral alterations between adult and middle-aged mice. In both WT and KO mice, we observed significant shifts in correlations suggesting a transition in the dependence of strategy usage as mice age (Fig. 6*A*,*B*). WT middle-aged mice overall had reduced use of the serial strategy and adopted the inefficient random search strategy, which was confirmed by a strong significant correlation between spatial and serial strategy use in adult WT (Fig. 6*A*, top) and significant correlations between spatial and random strategies in middle-aged WT mice (Fig. 6*B*, top). Although distinct patterns between adult and middle-aged mice emerged in both genotypes, the patterns in middle-aged KO mice were highly like those of adult WT mice, where the correlation between spatial and serial strategy use was strongest (Fig. 6*B*, bottom). Cumulatively, these findings suggest that middle-aged *Rnf216* KO mice exhibit strategies that are more tuned to adult WT mice, which could reflect a developmental stunting of strategy formation. Overall, we conclude that middle-aged KO mice have a breakdown in performance necessary to support the use of age-appropriate strategies. Moreover, middle-aged *Rnf216* KO mice begin to develop subtle fear learning impairments, which is concordant with the development of dementia in individuals with GHS (Margolin et al., 2013; Alqwaifly and Bohlega, 2016).

### *Rnf216* KO males display lower intrinsic excitability in hippocampal CA1 neurons

**Figure 7.**
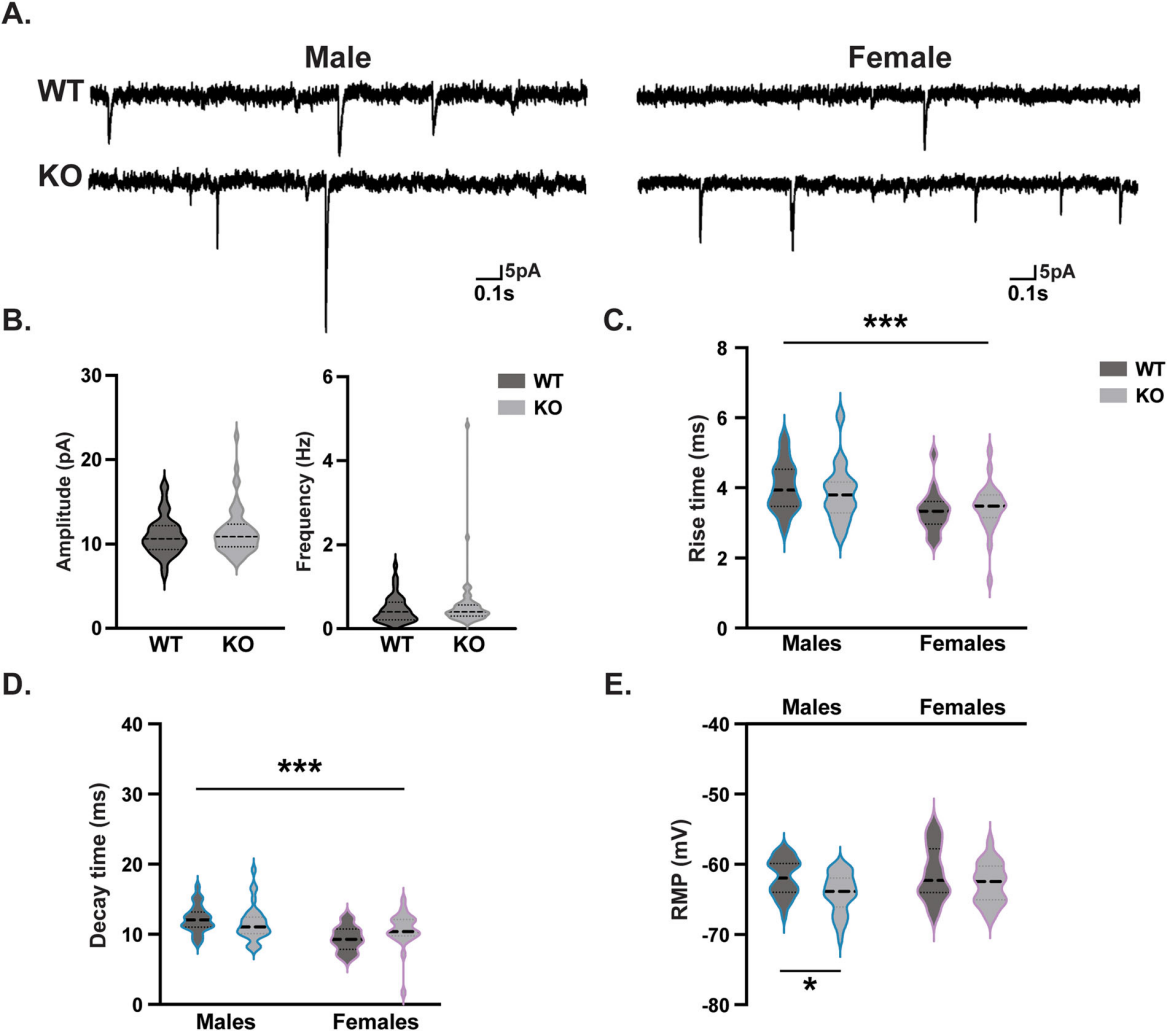
Decreased intrinsic excitability in the hippocampus of *Rnf216* KO male mice. ***A***, Representative miniature excitatory postsynaptic potential (mEPSP) traces from WT and KO male and female mice. There were no differences in (***B***) amplitude or frequency. ***C***, Sex differences were observed in rise time. *F*_Sex(1,94)_ = 13.45, ***p = 0.0004; *F*_Genotype(1,94)_ = 0.04758, *p* = 0.8278; *F*_Sex*Genotype(1,94)_ = 0.8068, *p* = 0.3714. Post hoc: WT-Males vs WT-Females, *p = 0.0181; WT-Males vs KO-Females, *p = 0.0262. Two-way ANOVA with Tukey’s multiple comparisons. ***D***, There were sex differences in decay time. *F*_Sex(1, 94)_ = 15.77, ***p = 0.0001; *F*_Genotype(1,94)_ = 0.4680, *p* = 0.4956; *F*_Sex*Genotype(1,94)_ = 3.839, *p* = 0.0530. Post hoc: WT-Males vs WT-Females, **p = 0.0011; KO-Male vs WT-Female, *p = 0.0109. Two-way ANOVA with Tukey’s multiple comparisons. ***E***, More negative RMP in KO male mice. *F*_Sex(1, 94)_ = 2.363, *p* = 0.1276; *F*_Genotype(1,94)_ = 8.877, **p = 0.0037; *F*_Sex*Genotype(1,94)_ = 0.4267, *p* = 0.5152. Post hoc: WT-Males vs KO-Males, *p = 0.0258; KO-Males vs WT-Females, *p = 0.0144. Two-way ANOVA with Tukey’s multiple comparisons. *N* = 4 mice per sex/genotype and *n* = 16 cells for WT-Females, 24 for KO-Females, 37 for WT-Males, and 43 for KO-Males.

Due to the role of RNF216 in synaptic plasticity and receptor trafficking (Mabb et al., 2014; Wall et al., 2018; George et al., 2022) and our findings related to fear conditioning, we sought to determine if neurotransmission was altered in *Rnf216* KO mice. We measured mEPSCs in CA1 neurons of acute hippocampal slices (Fig. 7*A*) in P28 mice. In both males and females, there were no differences in amplitude or frequency (Fig. 7*B–D*); however, there were sex differences in rise and decay times (Fig. 7*C*,*D*). Notably, *Rnf216* KO males had a more negative RMP (Fig. 7*E*), suggesting a decreased probability of firing action potentials in male KO mice. There were no significant differences observed in females.

### Adult *Rnf216* KO mice have altered microglia in the hippocampus and cortex

**Figure 8.**
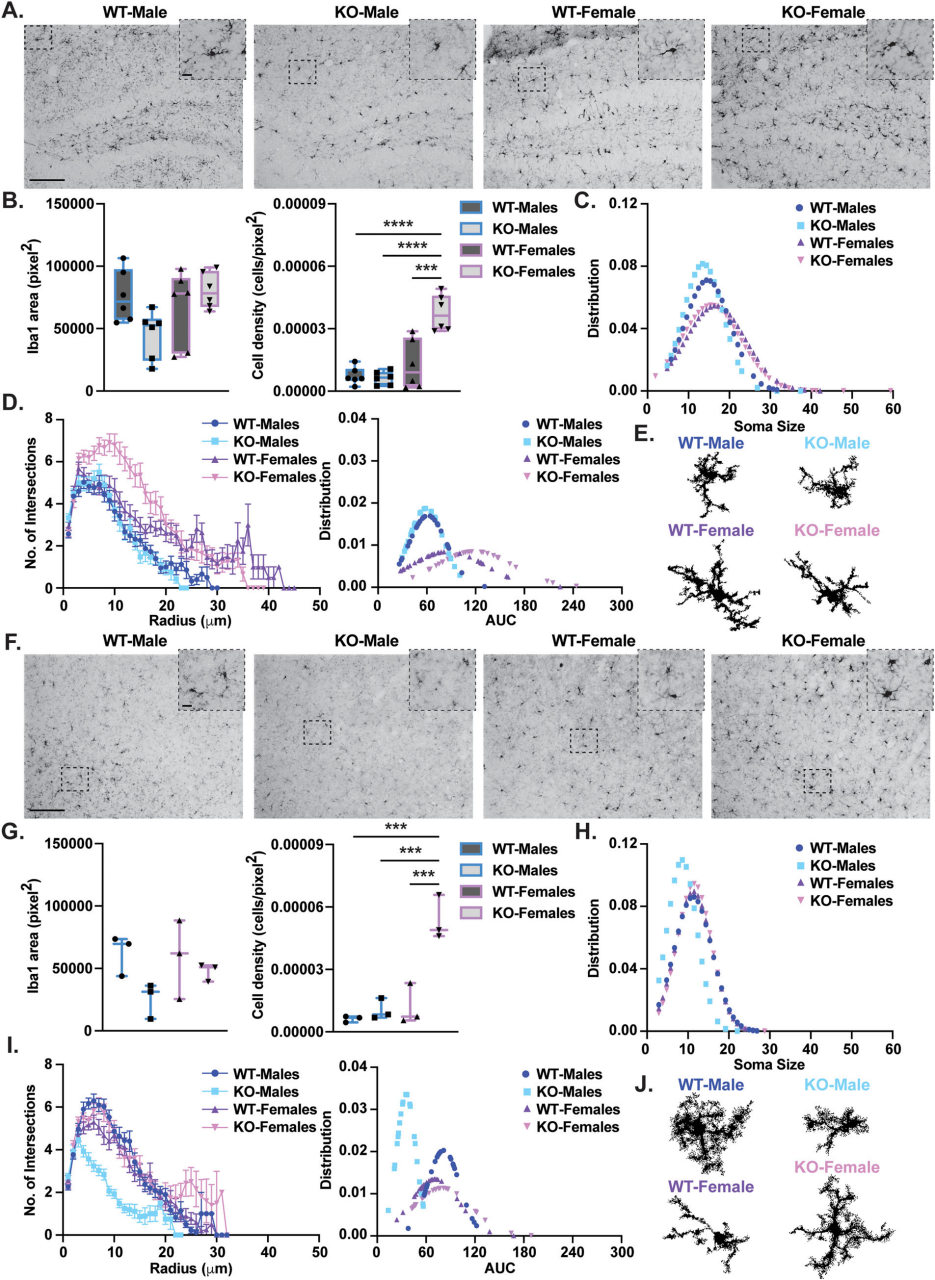
Altered microglia in the hippocampus and cortex of adult *Rnf216* KO mice. ***A***, Top, Representative images of microglia stained with Iba1 in the hippocampus in adult (∼P112) WT and KO males (left) and females (right) imaged at 20× magnification. Scale bar represents 100 µm, inset represents 10 µm. ***B***, Left, Area of Iba1. *F*_Sex(1,20)_ = 2.087, *p* = 0.1641; *F*_Genotype(1,20)_ = 0.9083, *p* = 0.3519; *F*_Sex*Genotype(1,20)_ = 6.251, *p = 0.0212. Two-way ANOVA. Right, Density of Iba1-positive cells. There were significant increases in cell density but not area in KO-Females. *F*_Sex(1,20)_ = 33.08, ****p < 0.0001; *F*_Genotype(1,20)_ = 14.13, **p = 0.0012; *F*_Sex*Genotype(1,20)_ = 16.69, ***p = 0.0006. Post hoc: WT-Males vs KO-Females, ****p < 0.0001; KO-Males vs Female KO, ****p < 0.0001; WT-Females vs KO-Females, ***p = 0.0001. Two-way ANOVA with Tukey’s multiple comparisons. *N *= 3 per sex/genotype with 1–2 sections per mouse represented in summary plots. ***C***, Distribution of soma size in WT and KO mice. Male KO mice have smaller soma size. Welch’s *t* values for WT-Male vs KO-Male −0.472 (*p* = 0.033), WT-Male vs WT-Female −4.620 (*p* < 0.001), KO-Male vs KO-Female −12.116 (*p* < 0.001). *N* = 127–293 cells per genotype/sex. ***D***, Left, Sholl analysis of reconstructed microglia measuring number of intersections in proximity to the soma. Right, AUC fittings using hierarchical bootstrapping for individual microglia. Female microglia had more intersections than male microglia. Welch’s *t* value for WT-Male vs WT-Female 7.071 (*p* < 0.001) and KO-Male vs KO-Female 6.752 (*p* < 0.001). *N* = 3 mice per genotype/sex, *n* = 30 reconstructed cells per group. Error bars are represented as ± SEM. ***E***, Example 3D reconstructions of microglia from each group. ***F***, Top, Representative images of microglia stained with Iba1 in the cortex in adult (∼P112) WT and KO males (left) and females (right) imaged at 20× magnification. Scale bar represents 100 µm, inset represents 10 µm. ***G***, Left, Area of Iba1. *F*_Sex(1,8)_ = 0.6448, *p* = 0.4452; *F*_Genotype(1,8)_ = 4.515, *p* = 0.0663; *F*_Sex*Genotype(1,8)_ = 1.305, *p* = 0.2863. Two-way ANOVA. Right, Density of Iba1-positive cells. There were significant increases in cell density but not area in KO females. *F*_Sex(1,8)_ = 29.89, ***p = 0.0006; *F*_Genotype(1,8)_ = 26.11, ***p = 0.0009; *F*_Sex*Genotype(1,8)_ = 17.33, **p = 0.0032. Post hoc: WT-Males vs KO-Females, ***p = 0.0003; KO-Males vs KO-Females, ***p = 0.0006; WT-Females vs KO-Females, ***p = 0.0008. Two-way ANOVA with Tukey’s multiple comparisons. *N *= 3 per sex/genotype with 1 section per mouse represented in summary plots. ***H***, Distribution of soma size in WT and KO mice. Male KO mice have smaller soma size. Welch’s *t* values for WT-Male vs KO-Male 5.663 (*p* < 0.001) and KO-Male vs KO-Female −7.267 (*p* < 0.001). *N* = 128–267 cells per genotype/sex. ***I***, Left, Sholl analysis of reconstructed microglia measuring number of intersections in proximity to the soma. Right, AUC fittings using hierarchical bootstrapping for individual microglia. All groups were significantly different from one another with KO male microglia exhibiting the greatest reduction in intersection number. Welch’s *t* value for WT-Male vs KO-Male −4.330 (*p* < 0.001), WT-Female vs KO-Female 2.029 (*p* = 0.04), WT-Male vs WT-Female 3.267 (*p* = 0.002), KO-Male vs KO-Female 8.398 (*p* < 0.001). *N* = 3 mice per genotype/sex, *n* = 30 reconstructed cells per group. Error bars are represented as ±SEM. ***J***, Example 3D reconstructions of microglia from each group.

RNF216 has a function in innate inflammatory signaling (Chuang and Ulevitch, 2004; Fearns et al., 2006; Nakhaei et al., 2009) and phenotypes related to neuroinflammation were previously observed in the preoptic area of the hypothalamus of adult *Rnf216* KO male mice (George et al., 2022). We analyzed microglia density, area, soma size, and morphology in the dorsal hippocampus and S1 cortex of adult *Rnf216* WT and KO mice, by labeling microglia with the microglia-specific marker Iba1. Although there were no sex or genotypic differences in Iba1 total cell area (Fig. 8*B*, left), we observed a significant increase in the density of microglia selectively in *Rnf216* KO female mice (Fig. 8*B*, right). We further identified sex differences between WT male and female microglia soma size and a sex-specific reduction in male KO soma size (Fig. 8*C*). When evaluating microglia morphology using the Sholl-based method, sex differences in male and female microglia were observed with no genotypic differences. Overall, female WT and KO microglia at this age had an increase in the number of intersections compared with males (Fig. 8*D*,*E*). Surprisingly, there were no significant differences between WT and KO male microglia (Fig. 8*D*,*E*). When evaluating microglia in the S1 cortex (Fig. 8*F*), there were no differences in Iba1 area (Fig. 8*G*, left). Like the hippocampus, KO females had a selective increase in microglia density (Fig. 8*G*, right). Significant decreases in soma size were still observed in male KO mice, but there were no sex differences (Fig. 8*H*). Upon inspection of microglia morphology using Sholl analysis, there were significant changes between all groups (Fig. 8*I*,*J*), with male KO mice exhibiting a dramatic reduction in the number of intersections (Fig. 8*I*,*J*). Taken together, our findings indicate that global deletion of *Rnf216* leads to sex- and region-specific alterations in microglia characteristics in select brain regions. These alterations in microglia profiles over time may lead to proprioceptive phenotypes and increase susceptibility to cognitive disruptions observed in middle-aged GHS model mice.

## Discussion

In this study, we evaluated changes in motor and memory using *Rnf216* constitutive KO mice over the time course of a year. We speculated that deletion of *Rnf216* would result in motor and learning impairments that increase in severity upon age, similar to phenotypes observed in GHS patients (Margolin et al., 2013). In GHS, the onset of motor disturbances ranges in age from teens to middle adult age. Our 1-year-old mice are similar to middle-aged adults, which is why we selectively chose this time point for our behavior analysis (Dutta and Sengupta, 2016). However, to our surprise, we were unable to detect motor impairments or other measures of behavior that would be indicative of ataxia in these mice, which is a core feature of GHS (Gonzalez-Latapi et al., 2021). This lack of phenotype was also coupled with our inability to detect decreases in cerebellar mass that is a distinguishing feature in all late-stage GHS patients (Seminara et al., 2002; Margolin et al., 2013; Alqwaifly and Bohlega, 2016; Calandra et al., 2019; Chen et al., 2022). Both male and female *Rnf216* KO mice developed normal motor functions as demonstrated by righting reflexes and hindlimb clasping at 3 weeks of age. Deficits in the rotarod, open field, and grip strength tasks were also not observed, except for middle-aged KO mice traveling an increased distance in the center of the open field. However, *Rnf216* KO females did develop abnormal limb clasping beginning at 9 weeks of age, which affected a larger fraction of female KO mice at almost 1 year, suggesting that females may begin to develop proprioceptive deficits.

Surprisingly, adult *Rnf216* KO mice did not exhibit learning deficits in the Barnes maze spatial task, but middle-aged KO mice exhibited a bias in usage of a serial strategy across all task phases. Increases in serial strategy use were also observed in a previous transgenic mouse model for the RNF216 substrate Arc (Mabb et al., 2014; Wall et al., 2018). Here, mutation of RNF216-dependent Arc ubiquitination sites led to selective reversal learning deficits in juvenile male constitutive Arc knock-in mice (ArcKR; Wall et al., 2018). However, in contrast to our findings here, ArcKR mice had an increase in error number that was also correlated with enhanced perseverance ratios and increased serial strategy use in the reversal phase only. Reasons for these discrepancies could involve the age in which we conducted this task (ArcKR mice were tested at the juvenile stage, whereas *Rnf216* KO mice were tested in early and late adulthood), E3 ligase compensation for Arc ubiquitination, additional requirements of RNF216-dependent Arc ubiquitination sites, or involvement of other RNF216 substrates. When comparing strategies during aging, middle-aged WT mice exhibited an increase in random over serial strategy search. However, *Rnf216* KO mice exhibited serial strategy correlation profiles that were more similar to adult WT mice, suggesting that KO mice have delays in age-appropriate use of the random search strategy. These subtle differences in strategy use may be reflective of age-related changes in the output from the hippocampal system to associative areas such as the prefrontal cortex, which is selectively active during spatial navigation strategy and responsible for goal location and route planning (Poucet et al., 2004; Negrón-Oyarzo et al., 2018).

The fear conditioning task in our study had two components: context- and cue-dependent recall, both of which have overlapping and nonoverlapping microcircuits underlying each type of recall. Although there were no differences seen during cue recall, middle-aged *Rnf216* KO mice displayed decreased freezing time only during context recall. The underlying circuitry of fear conditioning includes projections from ventral hippocampal CA1 to the basal amygdala that encode context-dependent recall (Kim and Cho, 2020). However, during cue recall, some studies show that an auditory cue excites PV^+^ interneurons that indirectly disinhibit the basolateral amygdala (Wolff et al., 2014), while other studies posit that auditory stimuli evoke the pathway from the thalamus to the lateral amygdala (Bordi and LeDoux, 1994). From this, we gather that RNF216 may not directly impair whole brain regions but rather influence transmission between distinct brain regions. This is consistent with GHS clinical cases that show neuropathologies with loss of neurons and atrophy in specific brain regions and hippocampal inclusion bodies, indicative of neurodegeneration. Because there are multiple brain regions affected in GHS individuals, it is possible that the connections between them become dysfunctional.

The unique feature of this study compared with the clinical literature that identified specific *RNF216* mutations is that we used a constitutive KO model. Not all GHS patients have deletions, but rather point mutations that create dysfunctional RNF216 proteins (Margolin et al., 2013), which may account for the presence of a motor phenotype. However, male and female individuals with *RNF216* mutations that were predicted to be deleterious still exhibit ataxia, so using *Rnf216* KO mice is deemed an appropriate disease model. As an example, individuals with an E205fsX15+ C59X mutation or P606L mutation demonstrate severe limb, gait, appendicular, and truncal cerebellar ataxia with prominent cerebellar atrophy (Mehmood et al., 2017; Calandra et al., 2019). Homozygous variants predicted to be deleterious such as c.2251C>T and c.2149C→T found within the catalytic domain of RNF216 still display progressive ataxia with cerebellar atrophy (Margolin et al., 2013) indicating regardless of the type of mutation, ataxia is still present. Along with progressive ataxia, some individuals with heterozygous mutations of G13GfxX74 and C597X+E205DfsX15 that lie outside the catalytic domain also display chorea which involves involuntary jerking movements, distinct from ataxia. The human *RNF216* gene differs from mouse in that *RNF216* has five predicted isoforms (TRIAD3A, TRIAD3B, TRIAD3C, TRIAD3D, and TRIAD3E) that are invariably spliced at the N terminus (Chuang and Ulevitch, 2004), while mouse *Rnf216* has only three predicted isoforms (TRIAD3A, TRIAD3B, and TRIAD3C; Consortium, 2020). It is possible that although both humans and mice express TRIAD3A, TRIAD3B, and TRIAD3C, the additional human isoforms such as TRIAD3D/E (that are also differentially expressed in the brain) have a more dominant role in specific pathologies such as ataxia (Chuang and Ulevitch, 2004). Our *Rnf216* KO mouse is predicted to remove all three mouse isoforms of RNF216, but it is possible that these mice still express truncated versions of different isoforms. Our inability to detect any measure of brain volume loss in this study and in previous work (George et al., 2022) may point to additional distinct differences in how RNF216 is utilized in rodents compared with humans. This could in part be resolved by studying human models that include patient-derived iPS cells. Another possibility is that compound mutations in other genes in addition to *RNF216* may contribute to a more severe phenotype. Due to identification of digenic recessive mutations in GHS individuals that contain *OTUD4* and *RNF216* (Margolin et al., 2013), other unidentified genes may be participating in this complex syndrome. Environmental factors may also drive some of the severe phenotypes in human patients, which could be further pursued in our mouse model.

Another layer of data uncovered in this study is that electrophysiological and microglia alterations in constitutive male and female *Rnf216* KO mice were found to be region and sex specific. Functionally, microglia are involved in synaptic maturation through synaptic engulfment during postnatal development and are thought to be important for neuroprotection as a synaptic “stripper” (Trapp et al., 2007; Paolicelli et al., 2011; Kettenmann et al., 2013). It is known that microglia profiles differ between males and females (Schwarz et al., 2012; Bordt et al., 2020). For example, as early as P0, sex differences in microglia morphologies are observed in the CA1 hippocampus and the paraventricular nucleus of the hypothalamus (Schwarz et al., 2012). The pro-and anti-inflammatory cytokine profiles also differ between sex and region upon aging (Schwarz et al., 2012). Although males had lower RMPs and reduced microglia soma size in the hippocampus, females had no change in RMP but an increased density of microglia. The sex differences we have reported with microglia in the hippocampus are concordant to those reported in young adult mice (Schwarz et al., 2012; Bordt et al., 2020). Upon examination of microglia branching using Sholl, there were no differences in the number of intersections between WT and KO males. However, KO females had an increase in the number of intersections around the soma compared with WT females. It is possible that the select microglia alterations in male mice might lead to reduced RMP in this region with the alternative interpretation that these are unrelated observations. Microglia express a myriad of receptors that are classically involved in neurotransmitter and cytokine signaling (Kettenmann et al., 2011) and also act as active sensors in the CNS for “on” and “off” receptor-mediated signaling (Hanisch and Kettenmann, 2007). Therefore, it is possible that the hyperpolarized state observed in early male hippocampal neurons disrupt microglia receptor-mediated signaling, preventing their appropriate function throughout development. Compared with the hippocampus, adult male KOs displayed altered microglia branching (decreased intersections via Sholl analysis) and decreased soma size in the cortex. The collective alterations in microglia in these different brain regions reflect their diversity in function, namely, in the remodeling of neuronal circuits which may become more pronounced with age (Hanisch and Kettenmann, 2007). Cumulatively, these data indicate that removal of *Rnf216* differentially alters microglia states in male and female KO mice.

One account for microglia differences could also be related to alterations in gonadotropin and steroid hormone release. Gonadal hormones are thought to play a role in microglia sex differences (VanRyzin et al., 2018). Although we did not measure estradiol directly in these mice, previous work did not identify ovarian failure in KO female mice (George et al., 2022). However, our previous studies also demonstrated that male KO mice selectively have elevated baseline FSH levels and reductions in inhibin B compared with WT with signs of increased neuroinflammation as evidenced by alterations in microglia area and increases in the pro-inflammatory cytokine IL-1β (George et al., 2022). This is consistent with our current work showing males, in contrast to female KO mice, display reduced microglia soma size and branching. Importantly, testosterone gives rise to sexual differentiation in the vertebrate nervous system (Morris et al., 2004), and estradiol, which is aromatized from testosterone, has effects on hippocampal morphology (Lephart, 1996). Accordingly, there may be a lack of organizational effects in KO males during the perinatal or puberty time point leading to gonadal failure and altered hormonal release. This may also explain male-specific deficits in RMP. It is important to note that the electrophysiological and microglia-related phenotypes precede any of the learning deficits that we have observed. These early changes may alter neural circuit development and changes in microglia status, which collectively may render KO mice susceptible to behavioral alterations upon aging.

Taken together, our findings suggest that RNF216 may have region- and sex-specific effects on anatomical and behavioral outputs. These alterations may also be present in male and female GHS individuals, which have yet to be evaluated in the clinical setting. Our findings of microglia alterations also suggest that a neuroinflammatory environment supersedes any display of neurodegeneration or behavioral impairments. Currently, there are limited treatment options for GHS whose pathologies worsen over time. Our study points to possible targets for therapeutic intervention specific to activated or reactive microglia. Identifying mechanisms and substrates involved in RNF216 ubiquitination would help elucidate the cause of neuroinflammation and different aspects of behaviors. Likewise, restoring behavioral impairments in *Rnf216* KO with these targets would help shed light on interventions in other disorders with similar dysfunctions.

## Synthesis

Reviewing Editor: Karyn Frick, University of Wisconsin-Milwaukee Decisions are customarily a result of the Reviewing Editor and the peer reviewers coming together and discussing their recommendations until a consensus is reached. When revisions are invited, a fact-based synthesis statement explaining their decision and outlining what is needed to prepare a revision will be listed below. The following reviewer(s) agreed to reveal their identity: Lisa Taxier, Timothy Jarome. Note: If this manuscript was transferred from JNeurosci and a decision was made to accept the manuscript without peer review, a brief statement to this effect will instead be what is listed below.

This revised study provides an extensive characterization of motor/cognitive behaviors in WT mice and in a mouse model of Gordon Holmes syndrome across two age points. Additionally, the authors assess microglial expression and intrinsic excitability within the hippocampus of wild type/KO mice, providing evidence of subtle sex differences in electrophysiological kinetics. The manuscript is an interesting and well done, and a thorough analysis of Rnf216-related human disorder, which has not been well studied yet in the field. The reviewers felt that the authors thoroughly addressed previous reviewer concerns, particularly by adding sex as a covariate in statistical comparisons. A few concerns remain-although most are minor, there was a more significant question about validation of the transgenic mouse line as described below. Please address the following reviewer concerns:

A main concern is a perceived lack of validation for the transgenic mouse line. The entire success of the breeding is based on a single western blot image in Figure 1A, which is not very convincing. Notable is that the molecular weight of Rnf216 is 95kDa, which would put it as the band below where the authors are pointing with the arrow. While the Bethyl Labs antibody here tends to detect the protein at a higher molecular weight, the company reported weight is much higher than what the authors see here and, again, they refer to the lower band. Considering that there are largely no effects from the KO, it is critical that the authors provide clear, convincing confirmation that the breeding schedule did work as intended, preferably through converging evidence (RNA and protein).

Related, in Figure 1A are those western blot images from males or females? This is important because although Rnf216 is on an autosome, the breeding with Cre mutants could have resulted in different KO efficiency across sexes.

Justification needs to be provided for describing 12-month-old mice as "aged." Given that life span of most commonly used lab mouse strains is ~24-30 months, mice at this age are at best "middle-aged" and possibly even just "mature adult" (See Flurkey K, Currer JM, Harrison DE. Mouse models in aging research. IN: Fox JG, Barthold SW, Davisson MT, Newcomer CE, Quimby FW, Smith AL, eds. The Mouse in Biomedical Research, 2nd Edition. New York; Elsevier, 2007, Volume 3:637-72.).

The constant changing between showing both sexes or collapsing them in some graphs is confusing. Although it may make sense to collapse when no sex effect is observed, the reader is never able to see if a pattern even existed for sex before the collapsing of data. It would be better if all graphs had sex separated and the authors only collapses for the statistical analyses.

For Figure 2, a legend specifying what each color refers to would be helpful.

The microglia data in Figure 8 is sometimes referenced as Figure 7 in the Results section. Please correct this.

## Author Response

We thank the reviewers for their feedback on our submitted manuscript. We have done our best to address reviewer concerns and have provided comments below related to reviewer queries: Reviewer Comments This revised study provides an extensive characterization of motor/cognitive behaviors in WT mice and in a mouse model of Gordon Holmes syndrome across two age points. Additionally, the authors assess microglial expression and intrinsic excitability within the hippocampus of wild type/KO mice, providing evidence of subtle sex differences in electrophysiological kinetics. The manuscript is an interesting and well done, and a thorough analysis of Rnf216-related human disorder, which has not been well studied yet in the field. The reviewers felt that the authors thoroughly addressed previous reviewer concerns, particularly by adding sex as a covariate in statistical comparisons. A few concerns remain-although most are minor, there was a more significant question about validation of the transgenic mouse line as described below. Please address the following reviewer concerns: We thank the reviewer for this comment and provide more information related to validation of the transgenic mouse line below. A main concern is a perceived lack of validation for the transgenic mouse line. The entire success of the breeding is based on a single western blot image in Figure 1A, which is not very convincing. Notable is that the molecular weight of Rnf216 is 95kDa, which would put it as the band below where the authors are pointing with the arrow. While the Bethyl Labs antibody here tends to detect the protein at a higher molecular weight, the company reported weight is much higher than what the authors see here and, again, they refer to the lower band. Considering that there are largely no effects from the KO, it is critical that the authors provide clear, convincing confirmation that the breeding schedule did work as intended, preferably through converging evidence (RNA and protein). The predicted molecular weight of RNF216 is above 95 kDa and the RNF216 band we had pointed out in the previous version actually runs in between the 100-150 kDa marker on our SDS-PAGE gels. Using the Bethyl Labs antibody, there is a lower band that shows up underneath at the 95 kDa range, which we believe is a nonspecific band from the antibody. The size of the band that is lost in the KO animals is consistent with the detection of the molecular weight of a FLAG-tagged version of the two largest human isoforms of RNF216 (A, 923 amino acids and B, 866 amino acids), which run at ~125 kDa based on our previous manuscript (Figure S2A)1 . The amino acid content of these human isoforms are similar in size to reported mouse isoforms (853 and 910 amino acids). Unfortunately, the antibody reagents that we have available give very high background binding, which we agree is a potential confound when validating loss of RNF216 protein. It is important to note that we have previously validated this mouse line, which can be seen in supplemental Figure 2 in our previous publication, which had to be omitted from reference based on eNeuro's blinding process for reviewers2 . Our validation included genotyping to confirm the presence of the LoxP site upon breeding with CMV-Cre (Figure S2B) and validating a loss of RNF216 from the hypothalamus and gonads from female and male mice (Figure S2C)2 . To ensure that genotypes are correct, we give each animal a unique id #, genotype our animals prior to weaning and also re-sample our animals upon their termination. All tails pre- and post-experimentation are cross-checked and then archived in the lab 4oC for any necessary future genotyping needs/validations. We agree that the reviewer has pointed out a valid concern that we did not consider the possibility that an X-linked Cre may have differential effects in male and female mice. In our previous publication, we detailed the careful breeding scheme with CMV-Cre mice based on recommendations from Coleman et al3 that counterbalances for Cre on one allele (Figure S2A)2 . These mice were further bred to remove the presence of the X-linked CMV-Cre to avoid phenotypic confounds of X-linked Cre expression. Moreover, the original paper generating this mouse indicates that the Cre gene in this mouse line is expressed in all cells and prior to embryo implantation, which is a time in which X chromosome inactivation occurs in females4 . While this line is commonly used as a global deleter line, and to our knowledge has not been reported to have differential deletion effects in males and females, it is still a possibility. To address this issue, as requested, we measured RNF216 protein expression using a new RNF216 antibody from LS Biosciences in both male and female KO mice in hippocampus, cortex, and cerebellum and include the new western blots in Figure 1A. We also expand the molecular weight range in the representative immunoblots to show the range of bands that we have detected. Once again, we validated the loss of a ~120 kDa band using two separate RNF216 polyclonal antibodies (the image shown in Figure 1A is an RNF216 antibody from LifeSpan Biosciences and we have also shown this with an RNF216 antibody from Bethyl Labs). Both antibodies recognize the largest mouse protein isoforms (A and B)). Upon analysis, we did not find any significant differences based on sex (see statistical Table 1 for results). Therefore, we can conclude that the 120 kDa version of RNF216 is indeed equivalently deleted in KO males and females. It is important to note that there are two separate studies that have used a similar targeting strategy to delete Rnf216. The first strategy describes the knockout first allele, which is identical to our targeted design, with the exception that the mice were not bred to Flp recombinase deleter mice and still contained a LacZ cassette. The authors confirmed deletion via genotyping and western blotting (molecular weight sizes of the protein was not reported in this publication) and further found neuroendocrine phenotypes5 that were similar to our previously published work2 . The second study used CRISPR-Cas9 to create a mutation that would result in deletion of Rnf216. This study targeted exons 4 and 56 , which are similar exons that were targeted in our transgenic design2 . Western blots from this study using anti-RNF216 antibodies identified a band at ~120 kDa, which was absent in KO mice, similar to what we are finding. Overall, there were no sex differences in RNF216 expression in male and female KO mice. As stated above, RNF216 does have 3 predicted isoforms. The C isoform is the smallest (662 amino acids), lacks most of the N-terminal domain but retains the C-terminal catalytic RBR-RING domain that is a highly conserved in other RBR E3 ubiquitin ligases like PARKIN, HHARI, and HOIP. Therefore, this mouse isoform is extraordinarily difficult to detect with specificity by any available commercial antibodies. One potential caveat of this mouse line is that it does not completely eliminate all isoforms, most notably the C isoform since it cannot be detected. While these isoforms were considered in the knockout first design strategy, it is possible that this C isoform is still expressed. This is a limitation that we now describe in the Discussion section of the manuscript. Finally, we attempted to validate effective deletion of Rnf216 at the RNA level. This was done by isolating and extracting RNA from Rnf216 WT and KO brain tissue, generating cDNA using reverse transcriptase and amplifying Rnf216 off the cDNA product using qPCR. Sanger sequencing was also used to validate the specificity of the primers to amplify Rnf216. First and foremost, we were able to amplify regions outside of the predicted deleted exons in Rnf216 KO mice, which suggests that this design does not lead to nonsense mediated mRNA decay. It is most likely that the mRNA is produced but the protein is a partial product or is prematurely degraded. We did not identify the presence of a lower molecular band with increasing intensity in the KO condition suggesting that the truncated RNF216 A and B protein isoforms are likely degraded/destabilized. Related, in Figure 1A are those western blot images from males or females? This is important because although Rnf216 is on an autosome, the breeding with Cre mutants could have resulted in different KO efficiency across sexes. As stated above, we conducted our validation using male and female KO mice. Please see new Figure 1A for the western blot images. Justification needs to be provided for describing 12-month-old mice as "aged." Given that life span of most commonly used lab mouse strains is ~24-30 months, mice at this age are at best "middle-aged" and possibly even just "mature adult" (See Flurkey K, Currer JM, Harrison DE. Mouse models in aging research. IN: Fox JG, Barthold SW, Davisson MT, Newcomer CE, Quimby FW, Smith AL, eds. The Mouse in Biomedical Research, 2nd Edition. New York; Elsevier, 2007, Volume 3:637-72.). We have now clarified mouse age. The term aged was not meant to confer "old" age as we explained in the Discussion section. We have now clarified the P365 age to confer middle-age throughout the manuscript figures and text. The constant changing between showing both sexes or collapsing them in some graphs is confusing. Although it may make sense to collapse when no sex effect is observed, the reader is never able to see if a pattern even existed for sex before the collapsing of data. It would be better if all graphs had sex separated and the authors only collapses for the statistical analyses. The collapsing of sex in our graphs was a request by a previous reviewer, who thought it was distracting and difficult to parse through all the graphs when all were separated. We felt that it would be best to adhere to this requested layout as the figures and graphs are easier to follow. Instead, we have provided individual statistical reports (see Table 1) for all figures that were analyzed for sex differences. This includes those that were collapsed and those that remain in the manuscript. We felt that the report would allow readers to determine if patterns or trends may have existed as the differences are reported in the F statistics, which is the ratio of the two variances. For Figure 2, a legend specifying what each color refers to would be helpful. The legend is provided next to the value for each age on the right and we have added information on the figure related to sex. We have further clarified the color representations in the figure legend. The microglia data in Figure 8 is sometimes referenced as Figure 7 in the Results section. Please correct this. We apologize for this one type-o and have corrected this in the manuscript text. References: 1. Mabb AM, Je HS, Wall MJ, Robinson CG, Larsen RS, Qiang Y, Correa SA, Ehlers MD. Triad3A regulates synaptic strength by ubiquitination of Arc. Neuron. 2014;82(6):1299-316. doi: 10.1016/j.neuron.2014.05.016. PubMed PMID: 24945773. 2. George AJ, Dong B, Lail H, Gomez M, Hoffiz YC, Ware CB, Fang N, Murphy AZ, Hrabovszky E, Wanders D, Mabb AM. The E3 ubiquitin ligase RNF216/TRIAD3 is a key coordinator of the hypothalamic-pituitary-gonadal axis. iScience. 2022;25(6):104386. Epub 2022/05/28. doi: 10.1016/j.isci.2022.104386. PubMed PMID: 35620441; PMCID: PMC9126796. 3. Coleman JL, Brennan K, Ngo T, Balaji P, Graham RM, Smith NJ. Rapid Knockout and Reporter Mouse Line Generation and Breeding Colony Establishment Using EUCOMM Conditional-Ready Embryonic Stem Cells: A Case Study. Front Endocrinol (Lausanne). 2015;6:105. Epub 2015/07/16. doi: 10.3389/fendo.2015.00105. PubMed PMID: 26175717; PMCID: PMC4485191. 4. Schwenk F, Baron U, Rajewsky K. A cre-transgenic mouse strain for the ubiquitous deletion of loxP flanked gene segments including deletion in germ cells. Nucleic Acids Res. 1995;23(24):5080-1. Epub 1995/12/25. doi: 10.1093/nar/23.24.5080. PubMed PMID: 8559668; PMCID: PMC307516. 5. Melnick AF, Gao Y, Liu J, Ding D, Predom A, Kelly C, Hess RA, Chen C. RNF216 is essential for spermatogenesis and male fertilitydagger. Biol Reprod. 2019;100(5):1132-4. Epub 2019/01/17. doi: 10.1093/biolre/ioz006. PubMed PMID: 30649198. 6. Li D, Li F, Meng L, Wei H, Zhang Q, Jiang F, Chen DN, Li W, Tan YQ, Li JD. RNF216 regulates meiosis and PKA stability in the testes. FASEB J. 2021;35(4):e21460. Epub 2021/03/17. doi: 10.1096/fj.202002294RR. PubMed PMID: 33724554
